# Healthy Schoolhouse 2.0 Health Promotion Intervention to Reduce Childhood Obesity in Washington, DC: A Feasibility Study

**DOI:** 10.3390/nu13092935

**Published:** 2021-08-25

**Authors:** Melissa Hawkins, Sarah Irvine Belson, Robin McClave, Lauren Kohls, Sarah Little, Anastasia Snelling

**Affiliations:** 1 Department of Health Studies, College of Arts & Sciences, American University, Washington, DC 20016, USA; mcclave@american.edu (R.M.); stacey@american.edu (A.S.); 2 School of Education, American University, Washington, DC 20016, USA; sirvine@american.edu; 3 School of Public Affairs, American University, Washington, DC 20016, USA; lk9749a@student.american.edu (L.K.); sl6328a@student.american.edu (S.L.)

**Keywords:** childhood obesity, nutrition literacy, nutrition education, self-efficacy, teachers

## Abstract

Childhood obesity prevalence trends involve complex societal and environmental factors as well as individual behaviors. The Healthy Schoolhouse 2.0 program seeks to improve nutrition literacy among elementary school students through an equity-focused intervention that supports the health of students, teachers, and the community. This five-year quasi-experimental study follows a baseline–post-test design. Research activities examine the feasibility and effectiveness of a professional development series in the first program year to improve teachers’ self-efficacy and students’ nutrition literacy. Four elementary schools in Washington, DC (two intervention, two comparison) enrolled in the program (*N* = 1302 students). Demographic and baseline assessments were similar between schools. Teacher participation in professional development sessions was positively correlated with implementing nutrition lessons (*r* = 0.6, *p* < 0.001, *n* = 55). Post-test student nutrition knowledge scores (*W* = 39985, *p* < 0.010, *n* = 659) and knowledge score changes (*W* = 17064, *p* < 0.010, *n* = 448) were higher among students in the intervention schools. Students who received three nutrition lessons had higher post knowledge scores than students who received fewer lessons (*H*(2) =22.75, *p* < 0.001, *n* = 659). Engaging teachers to implement nutrition curricula may support sustainable obesity prevention efforts in the elementary school environment.

## 1. Introduction

The increasing prevalence of childhood obesity and overweight in the United States (US) is a significant public health concern, with adverse health and economic consequences across the lifespan [1]. Over the last two decades, the rates of childhood obesity increased from 13.9% to 19.3% nationally, and they disproportionately impacted communities of color and those of lower socioeconomic status [2]. In Washington D.C. (DC), the childhood obesity rate is among the highest in the nation, affecting 35% of children; in Wards 7 and 8, two of the district’s most underserved regions, the obesity rate is 72% [3]. The causes mediating childhood obesity prevalence involve complex societal and environmental factors, as well as individual behaviors; thus, solutions must engage multiple spheres of influence.

Several US federal policies have sought to address the system-wide challenges that contribute to an increase in childhood obesity and overweight within school settings. For example, the National School Lunch Program and Healthy, Hunger-Free Kids Act of 2010 (HHFKA) improved the nutrition standards school meals are required to meet [4,5], and the 2015 Every Student Succeeds Act (ESSA) included health and physical education in the definition of a “well-rounded education” [6]. Broad adoption of the Whole School, Whole Community, Whole Child (WSCC) model aligns education and health with a focus on the “long-term development and success of all children” [7]. Locally, DC passed the Healthy Schools Act of 2010 (amended by the Healthy Students Amendment Act of 2018) to establish comprehensive requirements for improving school meal and nutrition standards in the cafeteria and vending machines, increasing student physical activity and health education time, supporting school gardens and farm-to-school education, and establishing local school wellness policies to specifically address obesity and hunger [8].

Working in tandem with educational policy, health promotion interventions to improve child health and prevent obesity are frequently based in school settings. Although barriers exist, including time, resources, and educator training, extensive research supports the effectiveness of school-based nutrition education interventions to reduce childhood obesity and support academic achievement [9,10,11,12,13,14,15,16]. Specifically, health promotion interventions can improve nutrition knowledge, attitudes, and preferences for healthy foods [17,18] and serve to predict future behavior [19]. Further, a systematic review and meta-analysis of 34 studies [20] suggests a positive impact on student nutritional knowledge and dietary behaviors when nutrition education is taught by teachers.

Teachers are vital to advancing student health and education. Elementary school teachers, in particular, represent an upstream approach to establishing healthy eating habits early in life that may reduce future onset of childhood obesity. Moreover, investing in teacher knowledge and behavior can positively impact student outcomes [21]. While most teachers believe in the importance of teaching nutrition education and agree they can influence their students’ eating behaviors, less than half report feeling prepared, empowered, or able to integrate health education into their current curricula [22,23]. These findings [22,23,24] indicate that while teachers hold strong beliefs relative to the positive correlation between health and learning, they rate themselves poorly in having the knowledge and skills to teach and integrate health into instruction [25]. Accordingly, increasing teachers’ confidence, attitudes, and self-efficacy related to nutrition may have a positive impact on student health behaviors.

Healthy Schoolhouse 2.0 is a comprehensive childhood obesity prevention program that seeks to improve nutrition literacy among elementary school students and supports the health of students, teachers, and the community. The key component of the intervention is the empowerment of teachers as agents of change by equipping them with the skills, knowledge, and materials to integrate nutrition education in core subject areas [26]. The overarching approach of this work is based on the social ecological model (SEM) [27]. Additionally, it is grounded in the WSCC premise that recognizes it is more effective to establish healthy behaviors during childhood than to change unhealthy behaviors in adulthood that can result in overweight and obesity. This study examines the feasibility of the professional development (PD) program and impact of implementing nutrition lessons on students’ knowledge and attitudes among participants in their first year of the Healthy Schoolhouse 2.0 program. It is hypothesized that the student nutrition literacy scores are related to the number of professional development sessions teachers attended and number of nutrition lessons students received.

## 2. Materials and Methods

Healthy Schoolhouse 2.0 is a five-year feasibility study that follows a baseline posttest intervention design with staggered enrollment by study year. The data are multilevel in nature; teachers are nested within schools and students are nested within classrooms. The study methods are described previously in Hawkins et al. [26]. This analysis focuses on all student baseline and post-test scores during their first year of the program.

Teachers in the intervention schools participated in a five-session PD series designed to equip them with the skills, knowledge, attitudes, and materials to teach nutrition concepts within core subjects. The USDA’s *Serving up MyPlate Curriculum* provides nutrition education lessons that align with the common core standards in science, math, English language arts, and health [28]. Each PD session was offered in the schoolhouse, and teachers were invited to attend by the school principal, who also attended the sessions demonstrating leadership support for the topic and program. The sessions covered Healthy Schoolhouse 2.0 program and objectives, a socio-ecological approach to nutrition education, training on the *Serving up MyPlate: A Yummy Curriculum,* practice with model nutrition lessons, and nutrition myths and facts. Each session also included time devoted to address teacher health and wellness through mindfulness practices, stress-reduction techniques, breathing exercises, or gentle yoga poses. PD sessions were 30–45 min in length (offered in person at a time determined by school leadership), and teachers could then choose how many and which nutrition lessons they would implement, three serving as the criteria for program completion. Nutrition curriculum materials kits were created and delivered to teachers at each grade level in the intervention schools. In addition to the curriculum materials, all supplies needed to conduct lessons were provided in the kits. Technical support was offered to teachers in-person and online by the program manager. Teachers were asked to document each nutrition lesson implemented via a brief Google form. After implementing three lessons, teachers were eligible for a financial incentive that could be used to purchase classroom supplies. Students in the intervention and comparison schools completed a brief Student Nutrition Literacy Survey (SNLS) at baseline (*N* = 1302) and post to measure knowledge, beliefs, attitude, and intent toward healthful nutrition-related concepts. The SNLS was aligned with the USDA curriculum content. The design and validation of the SNLS instrument is described previously [29].

Approval for this study was obtained in July 2017 from the University’s Institutional Review Board for the Protection of Human Subjects in Research (IRB). Parent/guardian notification and assent was obtained in accordance with school district regulations through a written disclaimer that participation in the survey was optional. Student assent was obtained at the beginning of the school year prior to baseline survey data collection. Teacher consent was obtained at baseline.

### 2.1. Participant and School Demographics

Thirteen eligible schools in DC’s Wards 7 and 8 were invited to participate. Eligibility requirements included elementary schools that participate in the Community Eligibility Provision program, which serves breakfast and lunch at no cost to all enrolled students, provides instruction to students in grades 1–5, and has an active partnership with the community-based food access program. Schools were engaged via email invitation and phone calls from the program manager and then randomized to either the intervention or comparison group.

The populations at the four participating schools were statistically similar on several demographic covariates, including school size (<400 students), student ethnicity (>90% Black), and proportion of students eligible for free and reduced-price meals (100%). Table 1 describes the number of participating schools, students, and teachers.

### 2.2. Teacher Health Survey (THS)

Prior to participating in the PD series, teachers completed the previously administered [24] 38-item Teacher Health Survey (THS) regarding personal health habits, beliefs about health and education, and self-efficacy as it relates to implementing health-related content in the classroom. Health beliefs items (*n* = 8) (example item: “It is my role as a teacher to create classrooms that promote healthy habits for students.”) and self-efficacy items (*n* = 6) (example item: “I can motivate students to engage in healthy behaviors.”) were measured on a five-point Likert scale (1 “strongly disagree” to 5 “strongly agree”). Demographic information including teacher race/ethnicity and age were also collected. Biometric data (BMI) was not reported in order to reduce barriers to participation [30]. Therefore, it is not possible to examine if healthier teachers were more willing to participate in the program. Teachers’ interests in specific personal health and wellness topics were also incorporated in the PD sessions.

### 2.3. Student Nutrition Literacy Survey (SNLS)

The 15-item Student Nutrition Literacy Survey (SNLS) (Supplementary Figure A) was administered at baseline and post-intervention to students in the intervention and comparison schools. The SNLS has demonstrated appropriate initial psychometric qualities to measure nutrition knowledge, attitudes, and beliefs [29]. The instrument contains multiple choice questions with two scales that assess nutrition knowledge and attitudes, beliefs, and intent (ABI), with a KR20 of 0.7 for internal reliability for the knowledge items and 0.4 for ABI items. The survey is brief, easily administered, and has a low respondent burden. The SNLS incorporates the content of the USDA *MyPlate curriculum*, which is aligned with Common Core State standards in English and Math, as well as the Next Generation Science Standards, and the National Health Education Standards (Supplementary Table B). Each student’s name, grade, and gender were collected to assess changes in pre–post assessments at the individual level, although data were coded and de-identified during data entry. The SNLS was administered in-person by program graduate research assistants who read the questions aloud to students with consistent procedures in all classrooms. Students recorded their answers independently.

### 2.4. Data Analysis Procedures

Teacher and student survey data were entered, cleaned, and double-checked for accuracy by a different data coder before statistical analysis. Statistical analyses were performed using IBM SPSS Statistics software Version 26.0 (SPSS Inc., Chicago, IL, USA) and R ‘ordinal’ Package [31,32]. Descriptive analysis was conducted to summarize student and teacher sociodemographic characteristics, teacher self-efficacy and nutrition beliefs, and student nutrition literacy scores. Analysis was conducted on student level data which captures nutritional knowledge, attitudes and beliefs, teacher-level data and information on the PD sessions attended, and intervention lessons implemented within the classrooms.

For each scale on the SNLS, item responses were summed to create a scaled score with higher scores indicating positive nutrition attitudes. The SNLS mean score and standard deviation (SD) for each item, summary score for each scale, and percent correct score were compared between the intervention and comparison school at baseline and post. Skipped questions from the knowledge scale were marked incorrect; skipped questions from the ABI scale were excluded from the total score. Non-parametric analysis was conducted using Shapiro–Wilk’s Test, the Wilcoxon Signed-Rank Test, and the Kruskal–Wallis Test for data that were non-normally distributed and treated as ordinal in nature due to the discrete characteristics of the SNLS. Correlation analysis, using Pearson’s r correlation, was performed to examine the relationships among PD participation and lesson implementation. A Multilevel Mixed Linear Model using ordinal logistic regression (OLR) was used to determine the relationship between the predictor variables (intervention received, school type, gender, etc.) with the ordered factor dependent variable (SNLS knowledge percent scores) coded as ordered categorical grades A–F (A ≥ 90%, B ≥ 80%, C ≥ 70%, D ≥ 60%, F < 60%). In this case, OLR is more appropriate to use than linear mixed effects models because the SNLS score values are inherently categorical. Mean imputation, the replacement of a missing observation with the mean of the non-missing observations, was utilized upon independent variables with missing observations using the mice package in R [33].

## 3. Results

### 3.1. Teachers

**Figure 1:**
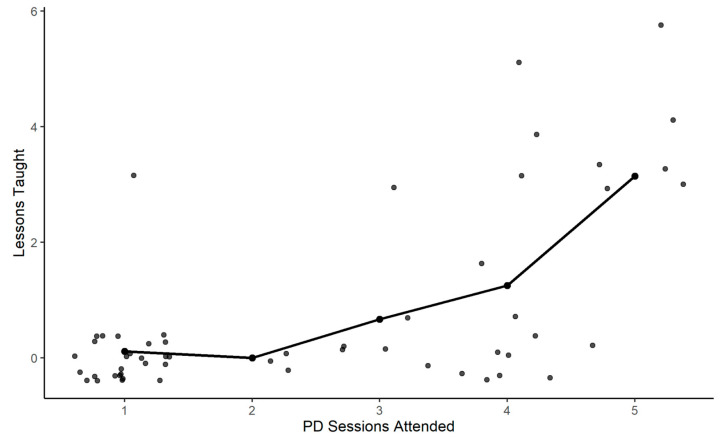
Correlation between professional development sessions and nutrition lesson implementation.

The majority of teachers identified as Black (66%) and female (90%) with a mean age of 41 years. Fifty-five teachers participated in one or more PD sessions; teachers attended an average of three of the five (SD = 1.5) PD sessions. Teachers implemented a total of 71 nutrition lessons. Among teachers who implemented lessons, the average number of lessons implemented was 4, with a range of one to nine lessons. There was a significant positive correlation between the number of PD sessions attended and the number of nutrition lessons implemented in the classroom (*r* = 0.6, *p* <0.01, *n* = 55) (Figure 1).

Teacher nutrition attitudes were positive overall in baseline and post-THS assessments among the intervention and comparison schools. There were no differences in the teacher pre-test self-efficacy scores and attitudes toward teaching nutrition between baseline and post survey administration (F(1,23) = 1.45, *p* > 0.050) or between intervention and comparison teachers (F (1,23) = 0.20, *p* > 0.050). There was no association between teacher baseline self-efficacy scores and participation in PD sessions among intervention school teachers (*r* (17) = 0.06, *p* = 0.98). It is important to note that baseline self-efficacy and nutrition attitudes mean scores were greater than 3, indicating that teachers generally agreed with the statements prior to the implementation of the PD sessions.

### 3.2. Student Nutrition Literacy Survey (SNLS)

A total of 1302 students in grades 1–5 completed the baseline SNLS. The baseline SNLS consists of pre-test and post-test scores for students in their first year of the Healthy Schoolhouse 2.0 program. Demographic characteristics were similar between students in the intervention and comparison schools (Table 2). Approximately 51% of students identified as male, and the sample was balanced by grade.

**Figure 2:**
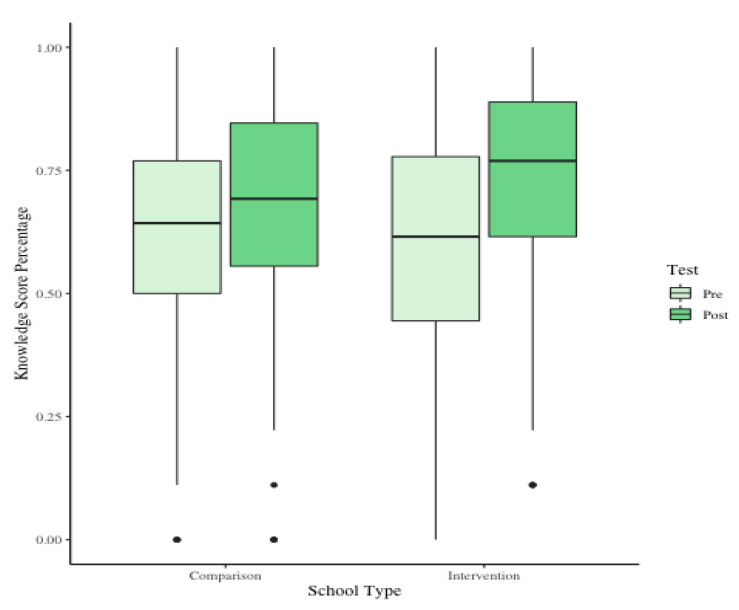
Distribution of student nutrition literacy knowledge scores in students first year of the program.

Baseline SNLS scores were compared using the Wilcoxon Ranked-Sum Test and were similar between intervention and comparison schools (W = 155929, *p* = 0.161). Both the intervention and comparison schools had an increase in median knowledge scores, from baseline to post measurements, 15.4% and 6.3%, respectively. Students in the intervention school experienced a significant increase in knowledge scores as demonstrated by the Wilcoxon Signed-Rank Test, which was the non-parametric alternative to a paired *t*-test (*p* < 0.001, *n* = 659). There was also a significant increase in knowledge percent score changes between students in the intervention and comparison schools (W = 17064, *p* < 0.001, *n* = 659) (Figure 2). Notably, students in the intervention schools had higher mean and median post scores on nutrition literacy knowledge than students in the comparison schools (W = 39985, *p* < 0.001, *n* =448). Student nutrition literacy knowledge scores were higher in higher grades overall, as expected. There were no statistically significant differences in median student attitude scores change by grade or between the intervention and comparison schools.

For the purpose of this analysis, the intervention was defined as having received three nutrition lessons (yes/no). A Kruskal–Wallis Test (H(2) = 22.75, *p* < 0.001, *n* = 659) and post hoc Dunn Test were utilized to examine if there were changes in SNLS scores with increases in the number (dose) of lessons. There was a significant difference between post-test scores of students who received the intervention of three nutrition lessons and those who received fewer lessons (0–2) (*p* < 0.001). Students who received three or more nutrition lessons had knowledge scores that were on average 10% higher than those students who received fewer lessons. Of note, there were no differences between the nutrition knowledge scores of students who received three lessons and those who received more than three lessons (*p* > 0.05).

### 3.3. Multilevel Ordinal Logistic Regression (OLR)

The hierarchical nature of school systems, with students nested within classrooms, lends itself to multilevel modeling approaches. Multilevel modeling allows for analysis of these hierarchical data using random effect variables. Random effects allow for random slopes to be added into the model to account for unobserved heterogeneity among schools (i.e., classrooms/schools). Odds Ratios are gathered by exponentiating OLR coefficients and indicate the odds of receiving a higher SNLS score given a 1-point increase in the predictor variable (e.g., nutrition lesson, gender, etc.). Table 3 describes the final OLR model. This model examined student SNLS scores for the first three years of the Healthy Schoolhouse 2.0 program and includes data for students in both the intervention and comparison schools. In this model, test exposure is operationalized as a time variable, beginning at zero, the students’ baseline test, and increasing incrementally each time a student takes the SNLS. The random variables of student ID, teacher ID, and test level were nested within school. Students who self-identified as female had higher SNLS scores than male students. The odds of receiving a higher score increased by 1.45 if the student was female. SNLS scores increased over time from pre to post assessment, with the odds of receiving a higher score increasing by 3.47. In the final OLR model, two interaction effects (test exposure and program year, and test exposure and test) were examined. Test exposure and program year examined the impact of student test exposure in different years of the Healthy Schoolhouse 2.0 program. For every one-unit increase in student test exposure times every unit increase in program year, there is a corresponding 0.68 increase in odds of a higher SNLS score. Test Exposure and Test examined the impact of the number of times a student sees the SNLS with the time of administration (pre or post). For every one-unit increase in student test exposure, the odds of a higher score on the SNLS post-test increases by 0.64.

**Figure 3:**
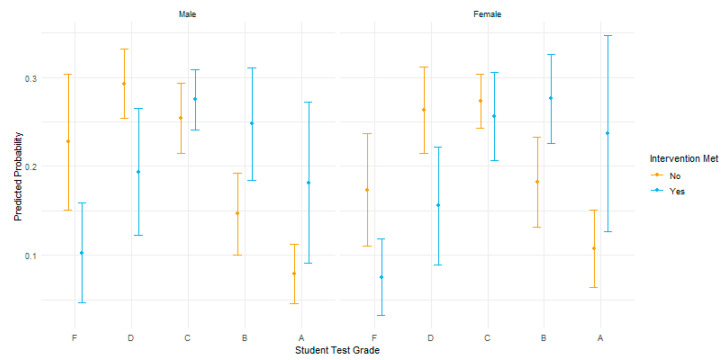
SNLS predicted grade probabilities by gender and intervention.

Figure 3 illustrates the predicted test grade probabilities by gender for students who received three or more nutrition lessons (intervention = yes) in blue and fewer than three nutrition lessons (intervention = no) in yellow. With all other variables held constant, students who received three or more nutrition lessons had a higher probability of scoring higher grades on the post SNLS assessment. Students who received three or more lessons have a higher estimated log odds of achieving >90% correct (A grade) on the post SNLS assessment than students who received less than three nutrition lessons. The odds of receiving a higher score (90% correct) over a lower score increased by 2.58 if a student received three nutrition lessons. It is important to note the clear downward trend of the log odds of the score predictions without nutrition lessons at a C grade. The predicted probabilities for students who received three or more nutrition lessons show a positive trend with higher probability of scoring higher nutrition literacy scores.

## 4. Discussion

Initial results from the participants in their first program year demonstrate that teacher participation in PD sessions and implementation of nutrition lessons has a positive association with increased student knowledge of nutritional concepts. Multilevel mixed modeling analysis demonstrated significant differences between pre and post nutrition knowledge scores between intervention and comparison schools, that the number of nutrition lessons implemented is significantly related to higher student knowledge scores, and there is a significant correlation between teacher PD sessions attended and number of lessons implemented in classrooms. In particular, students who received three nutrition lessons had significant improvement in nutrition literacy scores than students who received fewer nutrition lessons. This suggests that programs aimed to empower teachers as nutrition educators may be a valuable tool in teaching nutrition concepts and preventing childhood overweight and obesity within school systems.

The emphasis on supporting the “whole child” [7], including accountability for improving the health and wellness of students in the school environment, has been advanced with federal and state policies (e.g., HHFKA, ESSA) [34]. With the environment primed through policy levers to provide more nutritionally balanced meals, more dedicated time for physical activity and health education, and specific school wellness policy requirements to prevent and reduce obesity [35], the school setting is well positioned to support the nutrition literacy of students as well as the broader school community. Through improved nutrition literacy, students may be better equipped to increase their consumption of high-quality foods, which may in turn support overall physical health. Therefore, advancing nutrition literacy in the school setting is important to promote healthy eating and support long-term academic outcomes to reduce the burden of food-related diseases across the lifespan.

According to the principles of the social ecological model in which multiple spheres of influence must be mutually reinforcing, it is imperative to recognize the impact of teachers’ knowledge, engagement with PD, and implementation of nutrition lessons on students’ health and well-being outcomes [36] and equip them as agents of change in order for national and local policies to achieve desired aims. Previous research suggests that teachers offer a critical role in student motivation and engagement [37]. Teacher engagement is vital to influence student nutrition behavior and support efforts to reduce the prevalence of childhood obesity. The consistent contact that teachers, administrators, and staff maintain with students creates an opportunity to provide instruction and modeling of healthy nutrition patterns and other lifestyle habits and behaviors [24]. The preliminary results of Healthy Schoolhouse 2.0 support the feasibility of PD sessions with teachers on health-related constructs and content as a promising strategy to support obesity prevention efforts.

PD opportunities for teachers can help translate policy requirements into practical strategies and applications in the classroom. To be successful, professional learning events must both increase nutrition knowledge and also improve teacher confidence, attitudes, and self-efficacy in these areas. As previously described, research consistently supports the influence of teacher attitudes and self-efficacy [22,23,25,38]. Thus, to extend the influence of educators beyond their academic and curricular subject matter expertise, specific and appropriate training to increase knowledge in areas of health promotion and nutrition education is needed [39,40]. We plan to continue to focus on teacher engagement with PD in the remaining years of the project.

### 4.1. Strengths and Limitations

Healthy Schoolhouse 2.0 offers a feasible model for nutrition education programming and implementation, given the competing demands educators face. In the first year of the program, we learned that principal and/or assistant principal support is essential to the implementation of the program and to the engagement of the participating teachers. Teacher investment in 40–60 min of nutrition education over the course of the academic year may have a meaningful impact on student nutrition knowledge. While nutrition knowledge is only a single factor determining lifestyle habits in a complex environment of structural and cultural influences, it is an essential starting point for establishing lifelong health behaviors. In addition, the 5-year program provides students with progressive and cumulative instruction on key nutrition topics. Furthermore, the Healthy Schoolhouse 2.0 program implements the strategy of teachers acting as role models and being actively involved in the delivery of the intervention along with school policies that support the availability of healthy food.

There are several limitations to this study that are important to note. Although eligible schools were randomly allocated to the intervention or comparison groups, and all teachers at the intervention school were provided incentives to participate, engagement in the PD sessions at the teacher level was voluntary. However, the school setting is ideal for the implementation and evaluation of obesity prevention programs—in other health promotion programs, individuals who may be at high risk because of individual or environmental factors do not participate in such programs. The Healthy Schoolhouse 2.0 program provides the opportunity for equal participation by students regardless of nutrition risk factors. An equal number of intervention and comparison schools are enrolled, which allows for an analysis of multilevel intervention effects, in part because regression to the mean will impact the comparison schools as well as the intervention schools over the five-year study period.

We consider both clustering and nesting through multilevel modeling to account for the likelihood that students in each grade will be more highly correlated within a cluster than between clusters, and unique aspects of the clusters themselves may confound intervention effects. For example, Student’s *t*-test is predicated on the observations being statistically independent, the assumption that the data are normal and may underestimate standard errors, erroneously reducing *p*-values and increasing the risk of falsely rejecting a null hypothesis. Ultimately, after the five-year study period, we will have the opportunity to measure the stability of student nutrition knowledge and teacher self-efficacy over time as well as the long-term impact of the Healthy Schoolhouse 2.0 health promotion program.

There are additional threats to internal validity that are important to acknowledge given the quasi-experimental design. Student maturation is occurring over time that may be interpreted as an intervention effect, particularly when examining the impact of the Healthy Schoolhouse 2.0 intervention over the 5-year study period. Furthermore, student SNLS testing exposure may affect scores on subsequent assessments. There may be interactive effects of the Healthy Schoolhouse 2.0 intervention that may depend on the level of other nutrition interventions and efforts. Student consumption and food choice behaviors were not examined in this feasibility study. Future research is necessary to understand the association, if any, between student nutrition knowledge, attitudes, and healthful eating behaviors.

The results did not reveal baseline–post changes in teacher confidence in the first year of the program in either the intervention or the comparison schools. One explanation is a ceiling effect and decreasing variance from the baseline to post teacher survey. The small sample size may be an influencing factor in these findings, as many teachers did not complete both the pre and post surveys. A possible explanation for this low response rate for teachers may be the length of the teacher survey, which takes an average of 15 min to complete all sections. A dose–response of the lessons implemented in the intervention group is one potential explanation for the within-group results; however, an alternative explanation for this first-year association may be that those teachers who participated in the intervention school were more accepting of the intervention, open to teaching nutrition lessons, and different from teachers who did not implement lessons. Furthermore, this study measures secondary outcomes including nutrition literacy measures (knowledge, attitudes, behaviors) that are assumed to be the mediators of childhood obesity.

### 4.2. Implications for Future Research

Future research would benefit from exploring the relationship between school-level factors that we did not address, including how teacher knowledge may impact the integration of nutrition education into core classroom subjects: for example, identifying potential correlations between teacher self-efficacy to deliver lessons that incorporate nutrition content. Additionally, grade level may influence the ability to include nutrition education in lessons; specifically, lower grade levels may have more flexibility in their curricula because younger students are not required to participate in annual state standardized testing. A focus on intervention studies in early childhood settings would inform policies and practices to support early intervention and family engagement to reduce overweight and obesity in young children. Finally, longitudinal research on the distal effects of nutrition education on academic outcomes and obesity would validate claims about the benefits of policies and practices in schools.

## 5. Conclusions

Health behaviors established in childhood are critical determinants of health across the lifespan, particularly in regard to obesity prevention efforts [1]. From a public health perspective, the school setting represents great possibilities for advancing child health. The Healthy Schoolhouse 2.0 health promotion intervention program places teachers in a leadership role to support children’s nutrition literacy and health. This feasibility study addresses a need to support quality nutrition education in elementary schools. Furthermore, this study draws attention to the powerful role teachers can have on community obesity prevention efforts.

## Figures and Tables

**Table 1:** Healthy Schoolhouse 2.0 participants: Student baseline.

# Schools	# Students	Grades	# Teachers
4 (2 intervention, 2 comparison)	1302	1st–5th	55

#: Number.

**Table 2:** Student demographics: baseline assessment (*N* = 1302).

	Intervention (*n* = 694)		Comparison (*n* = 608)		Total (*n* = 1302)
	Intervention School 1	Intervention School 2	Comparison School 1	Comparison School 2	
	*n* (%)	*n* (%)	*n* (%)	*n* (%)	*n* (%)
Gender					
Male	202 (51.5%)	137 (45.4%)	157 (53.2%)	167 (53.4%)	663 (50.9%)
Female	174 (44.4%)	155 (51.3%)	128 (43.4%)	146 (46.6%)	603 (46.3%)
Not Reported	16 (4.1%)	10 (3.3%)	10 (3.4%)	0	36 (2.3%)
Grade					
1st	121(30.9%)	76 (25.2%)	93 (31.52%)	66 (21.1%)	356 (27%)
2nd	82 (20.9%)	53 (17.6%)	51 (17.29%)	60 (19.2%)	246 (18.9%)
3rd	55 (14%)	59 (19.5%)	50 (16.95%)	64 (20.4%)	228 (17.5%)
4th	68 (17.4%)	61 (20.2%)	51 (17.29%)	65 (20.8%)	245 (18.8%)
5th	66 (16.8%)	53 (17.5%)	50 (16.95%)	58 (18.5%)	227 (17.4%)

**Table 3:** Multilevel OLR analysis final model.

	Estimate	Odds Ratio	Std. Error	Z Value
Intervention (Yes)	0.95 ***	2.58	0.25	3.75
Test Exposure	1.2 ***	3.47	0.25	4.89
Program Year	0.39 ***	1.48	0.11	3.49
Test (Post)	1.5 ***	3.15	0.16	1.15
Gender (Female)	0.34 ***	1.45	0.11	3.11
Test Exposure and Program Year	0.38 ***	0.68	0.09	−4.13
Test Exposure and Test (Post)	0.44 ***	0.64	0.11	−4.127

*** Indicates *p* < 0.001.

## Data Availability

Healthy Schoolhouse 2.0 data are available upon request. The data presented in this study are available by request from the corresponding author.

## Supplementary Materials

The following are available online at https://www.mdpi.com/article/10.3390/nu13092935/s1, Supplementary Figure A: Student Nutrition Literacy Survey (SNLS), Supplementary Table B: USDA’s *Serving Up MyPlate: A Yummy Curriculum* Lessons.
