# Supplementary material for: Healthy Schoolhouse 2.0 Health Promotion Intervention to Reduce Childhood Obesity in Washington, DC: A Feasibility Study
Source: Nutrients. 2021 Aug 25;13(9):2935. doi: 10.3390/nu13092935 (PMC8464899; doi:10.3390/nu13092935)
Supplement: Supplementary file 1 [file nutrients-13-02935-s001.zip › Supplementary Figure A Student Nutrition Literacy Survey (SNLS).pdf]

Supplement A: Student Nutrition Literacy Survey[1] Level 2 (Grades 3-5)
Note: Level 1 Survey (Grades 1 & 2) includes only questions 1-14.

Name: ______________________________ Please circle: Boy Girl

Circle the best answer for each question.

1) Circle the most nutritious breakfast.

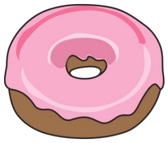
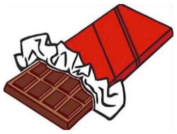
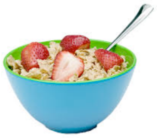

2) Circle the most nutritious drink.

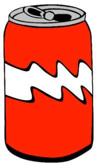
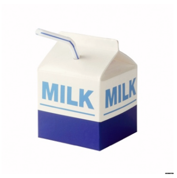

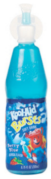

3) Circle the healthy snack.

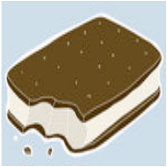
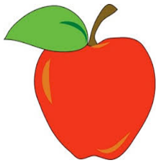
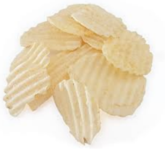

4) Circle the SOMETIMES food.

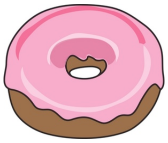
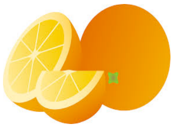
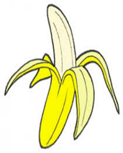

5) Circle the food group that builds strong bones with Vitamin D and Calcium.

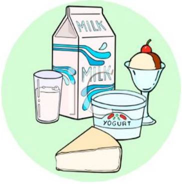

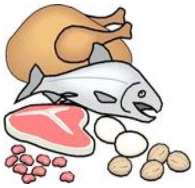
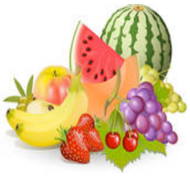

6) Circle the food group that heals cuts and scrapes with Vitamin C.

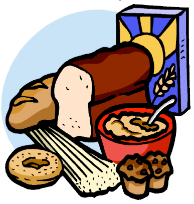
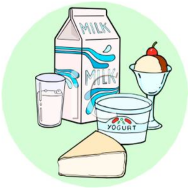

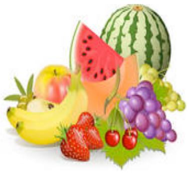

7) Circle the food group that builds muscles with protein.

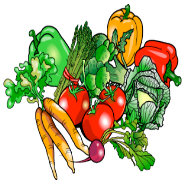 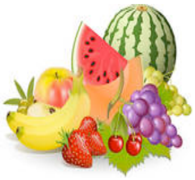 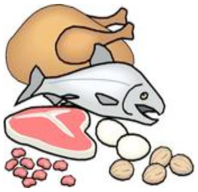

8) Circle the food group that helps eyes see better with Vitamin A.

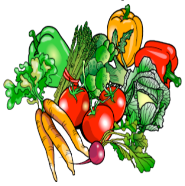 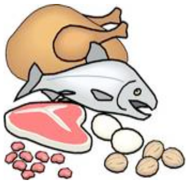 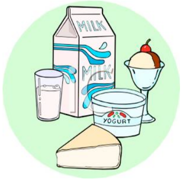

9) Circle the food group that provides the most energy with Vitamin B.

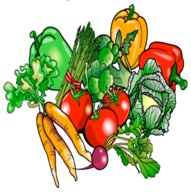 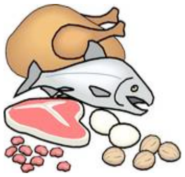 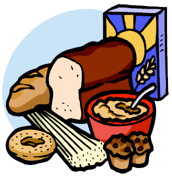

For the next 5 questions, circle yes or no.

10) I like to try new food.	Yes	No
11) I think fruit tastes good.	Yes	No
12) I like to eat vegetables.	Yes	No
13) I will try to drink water or milk instead of soda when I am not in school.	Yes	No
14) Physical activity makes me feel good.	Yes	No

15) Draw a line to match each taco ingredient to the correct food group. Tomato is matched to the fruit group as an example.

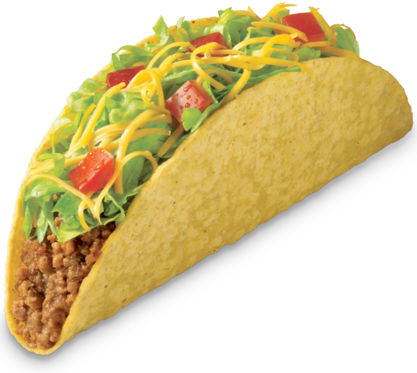

Tomato	Vegetable
Meat or beans	Grain
Lettuce	Fruit
Tortilla or taco shell	Dairy
Cheese	Protein

Adapted From the Fit Kids R Healthy Assessment - Gower, J. R., Moyer-Mileur, L. J., Wilkinson, R. D., Slater, H., & Jordan, K. C. Validity and reliability of a nutrition knowledge survey for assessment in elementary school children. *Journal of the American Dietetic Association*. 2010; *110*(3), 452-456.

1) Hawkins M, Fuchs H*, Watts E, Belson SI, Snelling A. Development of a nutrition literacy survey for use among elementary school students in communities with high rates of food insecurity. *Journal of Hunger and Environmental Nutrition*. 2021
