# Supplementary material for: Healthy Schoolhouse 2.0 Health Promotion Intervention to Reduce Childhood Obesity in Washington, DC: A Feasibility Study
Source: Nutrients. 2021 Aug 25;13(9):2935. doi: 10.3390/nu13092935 (PMC8464899; doi:10.3390/nu13092935)
Supplement: Supplementary file 1 [file nutrients-13-02935-s001.zip › Supplementary Table B USDAí»s Serving Up MyPlate A Yummy Curriculum Lessons.pdf]

Supplement Table. B: USDA's *Serving Up MyPlate: A Yummy Curriculum* Lessons.

	Lesson Title	Essential Question	Learning Objectives	Student Handouts
**Level 1**<br><br>**Grades 1 & 2**	**First Course:**<br>Fun with Food Groups<br><br>**Lesson 1, 2, 3**	What does it mean to be healthy? Eat healthy?	Students will be able to…<br>•Identify the five food groups and give examples of foods in each.<br>•Explain how MyPlate serves as a reminder to eat foods from each food group.<br>•Create a healthy meal containing a food from each food group.	1. My Food Card<br>2. My Menu Planner
**Standards Met***	**English Language Arts:** Speaking and Listening Standards: Comprehension and Collaboration (1.1, 2.1): Participate in collaborative conversations with diverse partners; Reading Standards: Foundational Skills/Fluency (1.4, 2.4): Read with sufficient fluency to support comprehension.<br>**Science:** Standard (A): Science as an inquiry: Understandings about Scientific Inquiry.<br>**Health:** Standard (1.2.1): Identify that healthy behaviors impact personal health.<br>**Math:** Number and Operations in Base Ten (1, 2): Use place value understanding and properties of operations to add and subtract.			
	**Second Course:**<br>Eat Smart to Play Hard<br><br>**Lesson 4, 5, 6**	Why is it important to eat a variety of foods from all food groups?	Students will be able to…<br>•Identify what foods to eat more of and explain why.<br>•Name at least two reasons why it is important to eat foods from all five food groups for a healthy diet.<br>•Explain that foods have nutrients that help us grow and stay healthy.<br>•Discuss how being physically active is part of a healthy lifestyle.	1. A Day in the Life of...<br>2. Serving MyPlate to My Family
**Standards Met***	**English Language Arts:** Speaking and Listening Standards: Comprehension and Collaboration (1.1, 2.1): Participate in collaborative conversations with diverse partners; Writing Standards: Production and Distribution of Writing (1.4, 2.4): Read with sufficient fluency to support comprehension.<br>**Science:** Standard (F): Science in personal and social perspective: Personal Health.<br>**Health:** Standard (1.2.1): Identify that healthy behaviors impact personal health.<br>**Math:** Number and Operations in Base Ten (1, 2): Use place value understanding and properties of operations to add and subtract.			
	**Third Course:**<br>Sometimes foods and Switcheroos	What foods should I eat less of, and why?	Students will be able to…<br>•Identify foods with added sugars and solid fats.<br>• Explain why foods with added sugars and solid fats should be eaten only some of the time.	1. "Sometimes" Foods and "Switcheroos"<br>2. "Switcheroo" Recipe

	**Lesson 7, 8, 9**		• Give examples of healthier food options to choose instead.	
**Standards Met***	**English Language Arts:** Reading Standards: Foundational Skills (1.4, 2.4): Read with sufficient accuracy and fluency to support comprehension. Speaking and Listening Standards: Comprehension and Collaboration (1.1, 2.1): Participate in collaborative conversations with diverse partners; (1.4, 2.4): Describe people, places, things, and events with relevant details, expressing ideas and feelings clearly; Writing Standards: Text Types and Purposes (1.2, 2.2): Write informative/ explanatory texts in which they name a topic, supply some facts about the topic, and provide some sense of closure.<br>**Science:** Standard (A): Science as an inquiry: Understandings about Scientific Inquiry; Standard (F): Science in personal and social perspective: Personal Health.<br>**Health:** Standard (5.2.1): Identify situations when a health-related decision is needed; Standard (5.2.1): List healthy options to health-related issues or problems. Standard (5.8.6): Choose a healthy option when making a decision.<br>**Math:** Measurement and Data (1, 2): Represent and interpret data; Number and Operations in Base Ten (1, 2): Use place value understanding and properties of operations to add and subtract.			
**Level 2**<br><br>**Grades 3 & 4**	**First Course:**<br>We Are What We Eat<br><br>**Lesson 1, 2, 3**	What choices can you make that help you stay healthy?	Students will be able to…<br>•Identify the five main food groups and name a variety of nutritious examples of foods in each.<br>•Explain how MyPlate serves as a reminder for how to eat a healthier meal.<br>•Create and describe a healthy meal containing a food from each food group.<br>•Discuss the importance of physical activity as part of a healthy lifestyle.	1. Who Am I? Clue Card 2. Food Writer
**Standards Met***	**English Language Arts:** Language Standards: Conventions of Standard English (3.2, 4.2): Demonstrate command of the conventions of standard English grammar and usage when writing or speaking; Vocabulary Acquisition and Use (3.5, 4.5): Demonstrate understanding of word relationships and nuances in word meanings; Conventions of Standard English (3.2, 4.2): Demonstrate reading standards for informational text (3.2, 4.2): Determine the main idea of a text and explain how it is supported by key details; summarizing the text; Writing Standards (3.1, 4.1): Write opinion pieces on topics or texts, supporting a point of view with reasons; Speaking and Listening Standards (3.1, 4.1): Engage effectively in a range of collaborative discussions with diverse partners, building on others' ideas and expressing their own clearly.<br>**Science:** Standard (A): Understandings about Scientific Inquiry: Classifying Objects.<br>**Health:** Standard (8.5.1): Encourage others to make positive health choices; Standard (6.2.1): Identify a short-term personal health goal and take action toward achieving the goal.			

	**Second Course:**<br>You Be the Chef<br><br>**Lesson 4, 5, 6**	Why is it important to eat a variety of foods from all food groups?	Students will be able to…<br>• Identify what foods to eat more of, and explain that nutrients in food help us grow and stay healthy.<br>• Name at least three reasons why it is important to eat foods from all five food groups for a healthy diet.<br>• Apply their knowledge of healthy foods and food groups to create a healthy meal or snack	1. Snack of Champions<br>2. Measuring Up MyPlate
**Standards Met***	**English Language Arts**: Speaking and Listening Standards (3.1, 4.1): Participate in collaborative conversations with diverse partners; Writing Standards (3.2, 4.2): Write informative/explanatory texts to examine a topic and convey ideas and information clearly.<br>**Health:** Standard (1.5.1): Describe the relationship between healthy behaviors and personal health; Standard (5.5.5): Choose a healthy option when making a decision; Standard (6.2.1): Identify a short-term personal health goal and take action toward achieving the goal.<br>**Science:** Standard (F): Personal Health: Nutrition is essential to health. Students should understand how various foods contribute to health.<br>**Math:** Numbers and Operations (3, 4): Fractions: Develop understanding of fractions as numbers			
	**Third Course:**<br>The Science of "Sometimes" Foods<br><br>**Lesson 7, 8, 9**	What are "sometimes" foods? Why are they called that? What can I eat instead?	Students will be able to…<br>• Identify foods that are high in solid fats and added sugars.<br>• Describe the benefits of limiting the consumption of solid fats and added sugars.<br>• Explain the concept of eating in moderation.<br>• Give examples of healthier food options to choose instead.	1. Experiment: Fats<br>2. Experiment: Added Sugars
**Standards Met***	**English Language Arts:** Reading Standards for Information Text (4.7): Interpret information presented visually, orally, or quantitatively (Meal 2); Writing Standards (4.2): Write informative/explanatory texts to examine a topic and convey ideas and information clearly.<br>**Science:** Standard (A): Understandings about Scientific Inquiry. Communicate investigations and explanations. Use data to construct a reasonable explanation. Use simple equipment and tools to gather data and extend the senses; Standard (F): Students should understand how the body uses food and how various foods contribute to health.<br>**Health:** Standard (1.5.1): Describe the relationship between healthy behaviors and personal health.<br>**Math:** Numbers and Operations (4): Use place value understanding and properties of operations to perform multi-d			
**Level 3 Grade 5**	**First Course:**<br>MyPlate,MySelf<br><br>**Lesson 1, 2, 3**	What choices can I make to be and stay healthy?	Students will be able to...<br>• Identify the five food groups and name a variety of examples from each.<br>• Explain how MyPlate serves as a reminder to eat from all five food groups.	1. MyPlate, MySelf

			• Create and describe a healthy meal containing foods from each food group ,including whole-grain options and a variety of vegetables.<br>• Discuss the importance of physical activity as part of a healthy lifestyle.	
**Standards Met***	**English Language Arts:** Language Standards: Conventions of Standard English, Vocabulary Acquisition and Use (5.1, 6.1): Demonstrate the command of the conventions of the standard English grammar and usage when writing or speaking; Speaking and Listening Standards: Comprehension and Collaboration (5.1, 6.1): Engage effectively in a range of collaborative discussions with diverse partners, building on others' ideas, and expressing their own clearly; Presentation of Knowledge and Ideas (5.4, 6.4): Report on a topic or text, or present an opinion. Writing Standards: Text Types and Purposes (5.2, 6.2): Write informative/explanatory texts to examine a topic and convey ideas and information clearly.<br>**Science**: Standard (A): Science as Inquiry: Understandings about Scientific Inquiry.<br>**Health:** Standard (8.5.1): Encourage others to make positive health choices; Standard (6.2.1): Identify a short-term personal health goal and take action toward achieving the goal.			
	**Second Course:**<br>Know Your Nutrients<br><br>**Lesson 4, 5, 6**	Why is it important to eat a variety of foods from all food groups?	Students will be able to…<br>• Identify the six main nutrients and a variety of foods that contain them.<br>• Explain how nutrients help us grow and stay healthy.<br>• Apply knowledge of healthy foods and food groups to create a healthy meal or snack.	1. Nutrient Knowledge
**Standards Met***	**English Language Arts:** Language Standards: Conventions of Standard English (5.1, 6.1): Demonstrate the command of the conventions of the standard English grammar and usage when writing or speaking; Speaking and Listening Standards: Comprehension and Collaboration (5.1, 6.1): Engage effectively in a range of collaborative discussions with diverse partners, building on others' ideas, and expressing their own clearly; Presentation of Knowledge and Ideas (5.4, 6.4): Report on a topic or text (5.5, 6.5): Include multimedia components (e.g., graphics, sound) and visual displays in presentations.<br>**Math:** Measurement and Data (5, 6): Represent and interpret data.<br>**Science:** Standard (F): Science in Personal and Social Perspectives: Personal Health.<br>**Health:** Standard (1.5.1): Describe the relationship between healthy behaviors and personal health; Standard (5.5.5): Choose a healthy option when making a decision; Standard (8.2.1): Encourage peers to make positive health choices.			
	**Third Course**:<br>Decisions, Decisions!<br><br>**Lesson 7, 8, 9**	What foods should I eat less of, and why? How can I make better choices?	Students will be able to…<br>• Identify ways to limit the consumption of solid fats, added sugars, and sodium.<br>• Read, compare, and analyze Nutrition Facts labels to determine which snack is a healthier alternative.	1. Nutrition Label Comparison<br>2. *Ad* Awareness

			• Summarize the benefits of limiting the consumption of solid fats, added sugars, and sodium.	
**Standards Met***	**English Language Arts:** Language Standards: Conventions of Standard English, Vocabulary Acquisition and Use (5.1, 6.1): Demonstrate the command of the conventions of the standard English grammar and usage when writing or speaking; Speaking and Listening Standards: Comprehension and Collaboration (5.1, 6.1): Engage effectively in a range of collaborative discussions with diverse partners, building on others' ideas and expressing their own clearly; Presentation of Knowledge and Ideas (5.4, 6.4): Report on a topic or text (5.5, 6.5): Include multimedia components (e.g., graphics, sound) and visual displays in presentations.<br>**Science:** Standard (A): Science as an Inquiry: Understandings about scientific inquiry; Standard (F): Science in Personal and Social Perspectives: Personal Health.<br>**Health:** Standard (1.5.1): Describe the relationship between healthy behaviors and personal health; Standard (8.5.1): Encourage others to make positive health choices			

* Sources: English Language Arts and Math standards—Common Core; Science education standards—National Academy of Sciences; Health standards—American Cancer Society.
